# EPIC-TABSAT: analysis tool for targeted bisulfite sequencing experiments and array-based methylation studies

**DOI:** 10.1093/nar/gkz398

**Published:** 2019-05-20

**Authors:** Julie Krainer, Andreas Weinhäusel, Karel Hanak, Walter Pulverer, Seza Özen, Klemens Vierlinger, Stephan Pabinger

**Affiliations:** 1 Austrian Institute of Technology, Center for Health & Bioresources, Molecular Diagnostics, Giefinggasse 4, 1210 Vienna, Austria; 2 Department of Pediatric Rheumatology, Hacettepe University, Hacettepe Hst., 06230 Ankara, Turkey

## Abstract

DNA methylation is one of the major epigenetic modifications and has frequently demonstrated its suitability as diagnostic and prognostic biomarker. In addition to chip and sequencing based epigenome wide methylation profiling methods, targeted bisulfite sequencing (TBS) has been established as a cost-effective approach for routine diagnostics and target validation applications. Yet, an easy-to-use tool for the analysis of TBS data in combination with array-based methylation results has been missing. Consequently, we have developed EPIC-TABSAT, a user-friendly web-based application for the analysis of targeted sequencing data that additionally allows the integration of array-based methylation results. The tool can handle multiple targets as well as multiple sequencing files in parallel and covers the complete data analysis workflow from calculation of quality metrics to methylation calling and interactive result presentation. The graphical user interface offers an unprecedented way to interpret TBS data alone or in combination with array-based methylation studies. Together with the computation of target-specific epialleles it is useful in validation, research, and routine diagnostic environments. EPIC-TABSAT is freely accessible to all users at https://tabsat.ait.ac.at/.

## INTRODUCTION

Epigenetic processes are biological mechanisms that alter the regulation of a gene without changing the DNA sequence. DNA methylation is the best characterized epigenetic modification (1) and occurs mainly in cytosine-guanine (CpG) dinucleotides when a methyl group is added to cytosine. In recent years, DNA methylation has demonstrated its suitability as biomarker and its potential for disease diagnosis and patient stratification (2–4). A powerful tool to detect DNA cytosine methylation is the treatment of the DNA with bisulfite, which converts unmethylated cytosine residues to uracil while leaving methylated cytosine residues unaffected (5). To detect this conversion, several methods have been developed. Two of the most prominent ones are *array-based methylation methods* (e.g. HumanMethylation450 BeadChips, Infinium MethylationEPIC BeadChip) and *bisulfite sequencing*. Illuminas Infinium platform is an array-based DNA methylation profiling technology that uses probes to detect up to 866.836 CpG sites in the human genome (6). Whole genome bisulfite sequencing (WGBS) is known as the *gold standard* for DNA methylation analysis as it allows investigation of all CpG sites at single-base resolution (7). Compared to other techniques, it is expensive, needs a large amount of input DNA to achieve sufficient read depth, and requires an analysis infrastructure capable of handling large amounts of data. In cases where epigenome wide studies are not required, *targeted bisulfite sequencing (TBS)* allows the analysis of specific loci while still retaining single CpG resolution. This strategy reduces the required amount of input DNA compared to WGBS. Sequencing can be performed on benchtop sequencers, which makes it a cost effective method with a quick turnaround time.

Several tools for the analysis of bisulfite sequencing data were already published, sharing common workflow tasks such as quality control, mapping the sequences to a reference, and extracting methylation information (see Supplementary Table S1). The main differences between the tools are the accepted number of target regions, the types of visualizations provided and the supported input data types. *BISMA* (8) takes data from sequencing subcloned molecules of PCR products (total number of sequence < 400), aligns it to one user specified reference and provides the methylation calling result in text format and as static plots. The web-based tool *QUMA* (9) also aligns reads against one given reference and outputs basic graphical representation of methylation patterns. Since both tools align against a single reference, they are not suitable for a TBS experiment with multiple regions. *BSPAT* (10) is available online and for download and specializes on the analysis and representation of methylation patterns. It generates static graphs that include a link to the UCSC genome browser to visualize the target regions. *Amplikyzer2* (unpublished) is a standalone software to analyze flowgrams and FASTQ files based on a specified target sequence, and outputs the methylation status and a basic plot. The latest addition to the BiQ Analyzer suite is *BiQ Analyzer HiMod* (11), a Java based analysis tool that requires separate input files for each sample-locus-treatment combination and outputs the detected methylation state. The recently published web-tool *BisAMP* (12) has been designed for RNA cytosine-5 methylation data processing. It displays graphically the results as heatmaps, and provides some functionality for the analysis of Illumina DNA-TBS. The only pipeline that focuses on interactive plots is *MethPat* (13), which skips the quality control and mapping steps and requires already existing methylation information as input.

Here, we present EPIC-TABSAT, an easy-to-use web-based tool for the integrated analysis of TBS data. It features an intuitive user interface, provides multiple outputs and visualizations, and offers an unprecedented way to analyze and interpret TBS data alone or in combination with array-based methylation studies. EPIC-TABSAT is suitable for use in research, clinical, and validation settings and is freely accessible at https://tabsat.ait.ac.at.

## WORKFLOW AND IMPLEMENTATION

**Figure 1.**
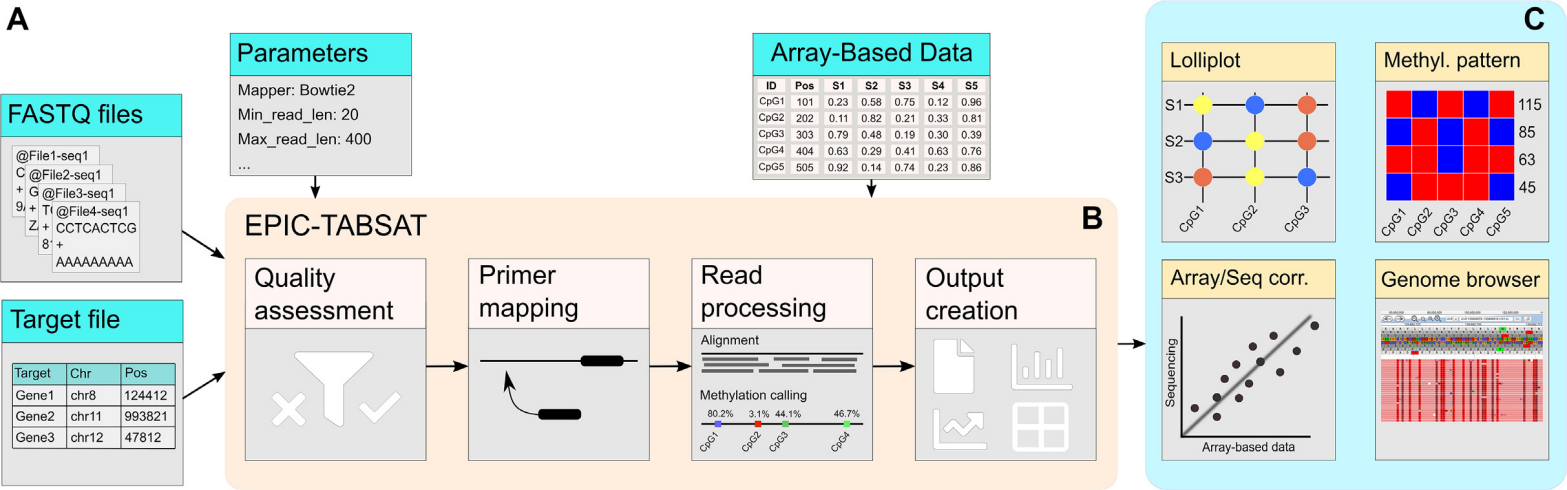
Workflow of the EPIC-TABSAT software. (**A**) Input to the software are FASTQ files, a file containing the targets of interest, and a set of parameters. Optionally, an array-based methylation data file can be provided which is combined with the sequencing data. (**B**) The workflow consists of several quality assessment steps, mapping, methylation calling, and output creation. **(C)** Methylation calling output and epiallele results along with quality metrics and the correlation of sequencing with the epigenome data are presented.

The EPIC-TABSAT workflow (see Figure 1) covers all required analysis steps from quality assessment to result presentation. Available outputs are, amongst others, interactive plots, statistics and a web-based genome browser view. Furthermore, in contrast to all other existing tools, it supports the combined analysis of TBS and array-based methylation data.

### File input

The graphical user interface offers an easy and intuitive way to upload data and start the analysis. Mandatory input files are the sequencing files in FASTQ(.gz) format, and a *target file* describing all regions of interest. Multiple samples can be analyzed in parallel by submitting several FASTQ files. EPIC-TABSAT works best when used with a medium number of targets (<150) and samples (<50) and supports a total file upload size of up to 10GB. Optionally, a multiple-sample methylation data file reduced to the targets of interest can be uploaded to perform a combined analysis of TBS and array-based data. Here, the data from different methods (e.g. Illumina Infinium platform, reduced representation bisulfite sequencing - RRBS) can be used and only need to be converted into the correct input format (see online help section). Optional adapter trimming can be performed by uploading a separate file containing the adapter sequences. EPIC-TABSAT provides various parameters to customize the analysis. Mandatory parameters are the reference genome (supported are hg19, hg38 and mm10) and the selection of the read mapping tool (Bowtie2 for Illumina; TMAP for IonTorrent). Eleven optional parameters can be set including minimum/maximum read length, minimum number of reads per CpG, minimum read quality, sequencing strategy, or restriction enzymes. EPIC-TABSAT provides a carefully selected default parameter-set and a sample dataset can be downloaded from the help page.

### Analysis workflow

The data analysis workflow of EPIC-TABSAT is based on the published tool TABSAT (14) and presents a major improvement. It contains the following novel features: (i) web-based interactive interface to start the analysis and view generated results; (ii) combined analysis of TBS and array-based methylation data; (iii) parallelization of processes to improve the analysis speed; (iv) support of the human reference version hg38; (v) display of restriction enzymes cut-sites; (vii) improved primer trimming and mapping and (viii) additional quality metrics and new visualizations. The current version of EPIC-TABSAT includes several improvements in response to filed issues made by external users on github.

The workflow starts with raw sequencing data, performs a quality assessment, and aligns the provided amplification primer sequences within the respective target region to the reference genome. This allows the assignment of the exact primer locations and is used to determine the exact target regions. Next, EPIC-TABSAT uses a tailored version of Bismark (15) to map the reads to the selected reference genome using all four DNA strands that result from bisulfite treatment. The mapping results are then used to extract the methylation state and reads spanning the individual target. Reads mapping outside of targets are also summarized. The software deduces specific methylation-patterns, which are aggregated and compared between samples. Finally, the results are combined and the HTML output is prepared.

### Backend

The backend of the EPIC-TABSAT workflow is a bash script, which calls multiple binaries, scripts, and tools using various programming languages (JAVA, Perl, Python, R). Apache is used to serve the web frontend, realized as a Python (2.7.6) Django (1.11.7) application based on Bootstrap (4.1.3). All applications used are open-source. EPIC-TABSAT runs on a 16-core Linux server with 148 GB of memory. The analysis of the sample dataset with five samples (∼3400 reads/sample) takes on average 19 min.

## OUTPUT AND VISUALIZATIONS

After submitting an analysis job, an unique ID is created and optionally sent per email. Using this ID, the status page can be accessed, which displays the used input parameters and the current progress of the analysis run. Upon completion of the job, a link to the results page is provided on the status page. The EPIC-TABSAT result view is structured into multiple tabs, which are designed with a strong emphasis on concise visualizations, summary metrics, and intuitive result presentation. The information contained in each tab is described in the following sections.

### Visualizations

EPIC-TABSAT provides two distinct primary visualizations of the results: a *lollipop* plot, and a *patternmap* figure. The graphs are largely based on the D3 library (https://d3js.org/) and plotly (https://plot.ly/) to support interactive presentation of information.

#### Lollipop

The lollipop plot is a graphical representation of the single CpG methylation status within each target for all samples. Groups of samples are separated by a red line. Using the lollipop plot options, the user can switch between targets, enable the display of previously defined restriction enzyme cut sites. Information about restriction enzyme cut sites can be used as a resource for additional experiments using methylation-sensitive restriction enzymes (MSRE). If array-based methylation data is supplied, the corresponding value will be displayed as an additional, color-dependent ring around the CpG circle. This unique combined visualization of sequencing and array-based methylation data allows users to easily compare and evaluate the two technologies. Below the lollipop graph, two boxplots are displayed to show groupwise methylation on CpG and target level.

#### Patternmap

**Figure 2.**
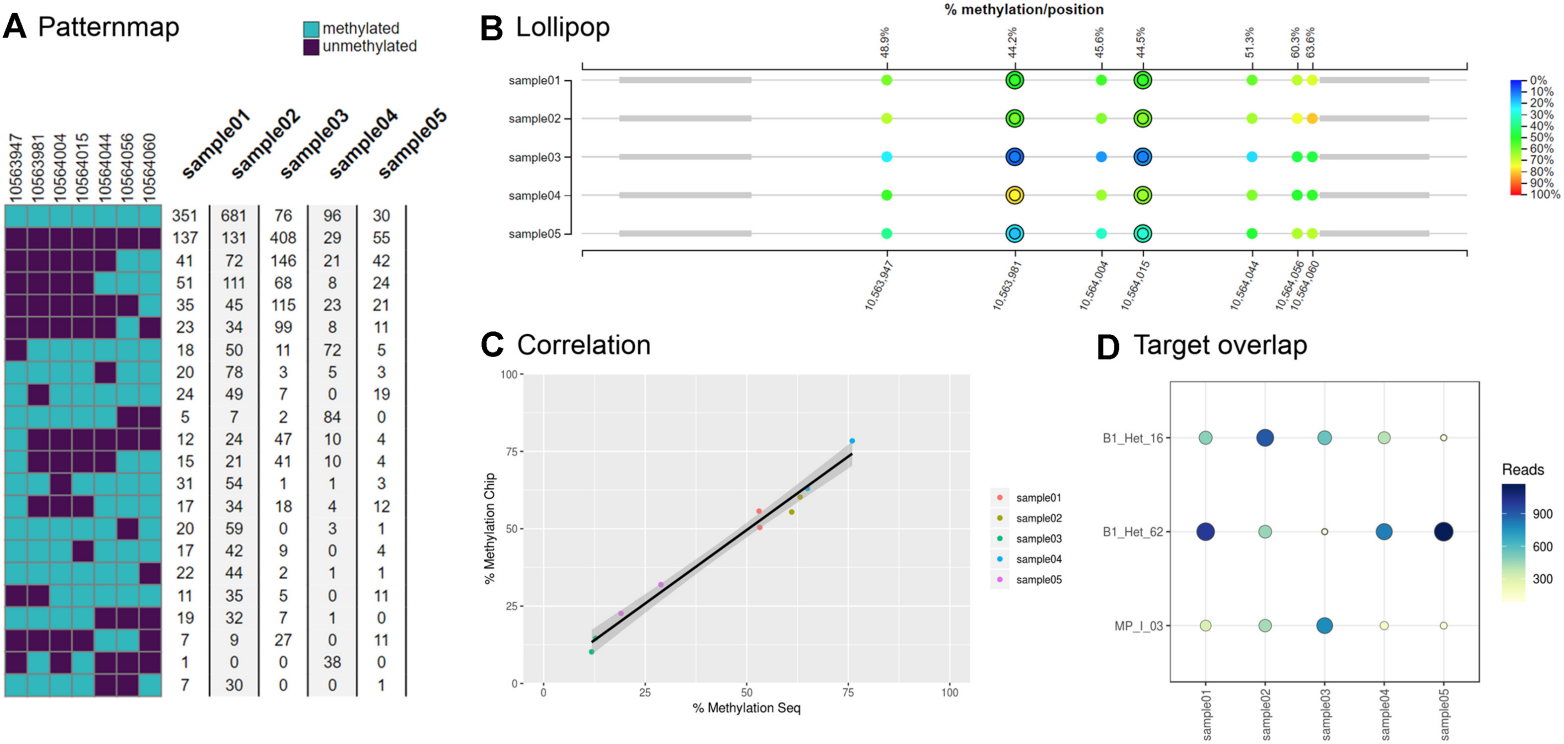
Overview of the graphical output of EPIC-TABSAT. (**A**) The different detected epialleles in the selected target region are shown in Patternmap and the number of alleles per sample is presented as a table. (**B**) The Lollipop plot shows the average methylation of each single CpG per sample and target. Rows represent samples and each single CpG is displayed as a circle spaced according to its actual chromosomal coordinate. The color of the circle indicates the percent methylation of the specific CpG site and primers are represented as gray boxes. The corresponding array-based data are displayed as an additional, color-dependent ring around the CpG circles. (**C**) The Correlation plot provides a visual representation of the correlation between sequencing and epigenome wide methylation data. (**D**) The number of reads spanning the whole target are shown in the Target overlap graph.

To get a detailed overview of available methylation patterns, called epialleles (16), the patternmap view (Figure 2A) shows the composition of methylation patterns present in the samples. Only reads spanning all CpGs within a target are considered and the distinct patterns are summarized for each sample. This allows the user to investigate whether specific samples have a higher abundance of epialleles compared to the other samples.

#### Array-sequencing correlation

The array-seq correlation page provides the user with two kinds of outputs. The overview table shows the average methylation of each CpG present in the targets along with the methylation-values of the array-based methylation experiment. These values are also presented as a scatter plot (Figure 2C). Furthermore, the associated pearson correlation coefficient is shown.

### Quality metrics

EPIC-TABSAT provides numerous metrics to evaluate the quality of the experiment. The summary view plots the coverage of each target for each sample as a heatmap. This allows a quick evaluation of the performance and confirming successful amplification of each target within each sample. Moreover, samples can be assigned to groups, which are used for generating boxplots. Furthermore, the quality control output of MultiQC (17) and PRINSEQ (18) are presented to evaluate read length- and quality distributions, mapping efficiency, and methylation calling performance. To inspect the alignment visually, a link to an integrated, web-based genome browser (19) is available, which automatically loads the selected reference, sample files, and target informations. This facilitates the identification of non-specific primers, wrongly specified targets, or pseudogenes. Displayed in the *off-target page* are regions, where reads mapped outside of the targets with a coverage above a user defined threshold.

#### Target-overlap

If the sequencing protocol allows for reads that span the whole target (from 5 to 3, or reverse), the target-overlap graph (Figure 2D) shows the count of these reads per sample for each target in graphical and table format.

#### Downloads

The generated methylation information can be downloaded as a table that reports the percent methylation as well as the number of methylated and unmethylated reads for all sample/CpG combination. This table can be used to perform further statistical analyses such as group comparisons. Additionally, FASTA files of the targets, BAM files, and methylation calling results created by Bismark are provided for all samples. A table containing the subclonal composition can be downloaded from the Patternmap section and plots can be stored in PNG format.

## EVALUATION AND DISCUSSION

EPIC-TABSAT is a comprehensive, user friendly, and feature rich analysis tool for targeted bisulfite sequencing data. It is a substantial improvement over the standalone tool TABSAT (14) and contains, amongst others, the following novelties: (i) a web-based, interactive user interface to start the analysis and view generated results; (ii) support of the combined analysis of TBS and array-based methylation data; (iii) significant internal method redesign to support intuitive result presentation, additional quality metrics, and a novel, sample specific methylation pattern analysis feature.

To evaluate the capability of EPIC-TABSAT, we analyzed the publicly available sequencing (SRR1951400) and epigenome wide methylation data generated in the study published by Yang et.al. (20). Although not originally designed to handle long-read sequencing data, EPIC-TABSAT was able to process the raw sequencing reads and generate the methylation calling output. The calculated correlation between sequencing and array-based methylation data is in line with the results reported by the original study.

Furthermore, we used IonTorrent sequencing data from a published study (21) that investigated the efficacy prediction for bevacizumab in breast cancer patients. The runtime for 32 samples (∼38k reads/sample) and 48 targets was 3.5 h.

EPIC-TABSAT offers several metrics for the evaluation of the quality of the experiment to proof the reliability of the generated results. In addition to raw read metrics, we also provide information about the target coverage and unspecific mapping to help with the identification of non-specific primers, incorrect target files, or pseudogenes. Due to the novel primer mapping functionality, the tool is able to correctly identify the amplified regions even if inaccurate targets were provided. Compared to other methylation analysis workflow applications, EPIC-TABSAT offers several different visualizations. As of right now, it is the only tool providing an end-to-end web-based analysis and visualization of targeted bisulfite sequencing studies in combination with array-based methylation studies. By joining these strategies, a powerful biomarker identification suite can be formed, where the array-based experiment acts as the discovery method followed by a validated study using TBS. Furthermore, TBS can be used to confirm biomarker candidates derived from public data or be used in a routine clinical setting to screen patients.

## OUTLOOK

EPIC-TABSAT has the potential to improve the analysis of targeted bisulfite data due to the incorporation of array-based methylation results and its intuitive user interface. The tool has been tested with inputs (>20) from different project teams using different sequencing technologies (Illumina, Ion Torrent, PacBio) and is online since September 2018. Due to the very positive feedback, we are constantly working on new extensions and features to improve EPIC-TABSAT further.

## Supplementary Material

gkz398_Supplemental_Files
