# Supplementary material for: EPIC-TABSAT: analysis tool for targeted bisulfite sequencing experiments and array-based methylation studies
Source: Nucleic Acids Res. 2019 May 20;47(W1):W166–70. doi: 10.1093/nar/gkz398 (PMC6602470; doi:10.1093/nar/gkz398)
Supplement: gkz398_Supplemental_Files [file gkz398_supplemental_files.pdf]

Tool	Targets	Mode	Reference	Data Input	Patternmap	Lollipop plot	QC plots	URL
Amplikizer2	multiple	Python application	custom ref	Flowgram; FASTA	no	static	no	pypi.org/project/amplikyzer2/
BiQ Analyzer HiMod	multiple	standalone; command line	custom ref	Sample-locusfile	static	static	no	biq-analyzer-himod.bioinf.mpi-inf.mpg.de/
BisAMP	multiple	web-based	custom ref	FASTQ	static	no	no	bisamp.dkfz.de/
BISMA	single	web-based	custom ref	ABI/FASTA	static	no	no	services.ibc.uni-stuttgart.de/BDPC/BISMA/
BSPAT	multiple	web-based	custom ref	FASTQ	UCSC track	no	no	cbc.case.edu/BSPAT/demo.jsp
EPIC TABSAT	multiple	web-based	hg19/38; mm10	Target File; FASTQ; Array file; adapter sequences	static	dynamic	yes	tabsat.ait.ac.at/
Methpat	multiple	command line	/	Bismark Methylation Output	interactive	no	no	bjpop.github.io/methpat/
QUMA	single	web-based	custom ref	Ref-Seq; Bisulfite Seq; FASTA	interactive	no	no	quma.cdb.riken.jp/

Supplementary Table 1: Comparison of tools for targeted bisulfite data analysis
